# Relationship of genetic polymorphisms in *CTLA-4* and *IL-18* with viral hepatitis: evidence from a meta-analysis

**DOI:** 10.1017/S0950268819001997

**Published:** 2019-12-05

**Authors:** Yang Yu, Jie Qu, Chen Zhou, Guangqiang You

**Affiliations:** 1 Department of General Surgery, China-Japan Union Hospital of Jilin University, Changchun 130033, Jilin, China; 2 Department of VIP Unit, China-Japan Union Hospital of Jilin University, Changchun 130033, Jilin, China; 3 Department of Personnel, The First Affiliated Hospital of Jilin University, Changchun 130000, Jilin, China; 4 Department of Hepatobiliary and Pancreatic Surgery, The Second Affiliated Hospital of Jilin University, Changchun 130041, Jilin, China

**Keywords:** *Cytotoxic T-lymphocyte-associated antigen 4* (*CTLA-4*), *interleukin-18* (*IL-18*), meta-analysis, polymorphisms, viral hepatitis

## Abstract

Relationship of genetic polymorphisms in cytotoxic T-lymphocyte-associated antigen 4 (*CTLA-4*) and interleukin-18 (*IL-18*) with susceptibility to viral hepatitis was already investigated by many association studies. The aim of this study was to more comprehensively analyse associations between genetic polymorphisms in *CTLA-4*/*IL-18* and viral hepatitis by combing the results of all relevant association studies. We searched Pubmed, Embase, Web of Science and CNKI for eligible studies. We used Review Manager to combine the results of eligible studies. Thirty-seven studies were finally included in this meta-analysis. Combined results demonstrated that *CTLA-4* rs231775 (recessive comparison: OR 1.31, 95% CI 1.11–1.55), *IL-18* rs1946518 (dominant comparison: OR 0.82, 95% CI 0.75–0.90; recessive comparison: OR 1.29, 95% CI 1.11–1.50; allele comparison: OR 0.76, 95% CI 0.68–0.86) and *IL-18* rs187238 (dominant comparison: OR 1.25, 95% CI 1.03–1.52; allele comparison: OR 1.20, 95% CI 1.05–1.37) polymorphisms were all significantly associated with viral hepatitis in the general population. Further subgroup analyses revealed that *CTLA-4* rs231775, *IL-18* rs1946518 and *IL-18* rs187238 polymorphisms were significantly associated with susceptibility to hepatitis B virus (HBV), especially among East Asians. Moreover, *CTLA-4* rs5742909, *IL-18* rs1946518 and *IL-18* rs187238 polymorphisms were also significantly associated with susceptibility to hepatitis C virus (HCV), especially among South Asians. So to conclude, this meta-analysis demonstrated that *CTLA-4* rs231775, *IL-18* rs1946518 and *IL-18* rs187238 polymorphisms may confer susceptibility to HBV in East Asians, while *CTLA-4* rs5742909, *IL-18* rs1946518 and *IL-18* rs187238 polymorphisms may confer susceptibility to HCV in South Asians.

## Introduction

Viral hepatitis refers to a group of infectious disorders caused by various kinds of hepatitis viruses (hepatitis A virus (HAV), hepatitis B virus (HBV), hepatitis C virus (HCV), hepatitis D virus (HDV) and hepatitis E virus (HEV)), and it could lead to life-threatening conditions including cirrhosis, liver failure or hepatocellular carcinoma [1, 2]. Although the exact mechanism of its pathogenesis is still uncertain, it was believed that genetic architecture was essential for the development of viral hepatitis. In the first place, the incidences of viral hepatitis in different populations vary greatly [3, 4], and genetic background was probably one of the reasons behind differences in disease prevalence across different populations. In the second place, previous association studies also identified numerous susceptible genetic loci of viral hepatitis [5, 6]. However, genetic factors that contribute to the development of viral hepatitis are still not fully elucidated.

Cytotoxic T-lymphocyte-associated antigen 4 (CTLA-4) and interleukin-18 (IL-18) are pro-inflammatory cytokines, and they both serve as crucial modulators of anti-viral immune responses [7, 8]. Therefore, if a genetic polymorphism could alter the transcription activity of *CTLA-4*/*IL-18* or the protein structure of *CTLA-4/IL-18*, it is biologically plausible that this polymorphism may also impact anti-viral immune responses and confer susceptibility to many types of infectious diseases including viral hepatitis.

In the past 20 years, results about associations between polymorphisms in *CTLA-4/IL-18* and viral hepatitis were already reported by many association studies, yet the conclusions of these studies were still inconsistent. To better analyse associations between polymorphisms in *CTLA-4/IL-18* and viral hepatitis, we carried out this study to get a more statistically reliable conclusion by combing the results of all relevant studies.

## Materials and methods

This meta-analysis was written in accordance with the PRISMA guideline [9].

### Literature search and inclusion criteria

To retrieve eligible articles, we searched Pubmed, Web of Science and Embase using key words listed below: (‘interleukin-18’ or ‘IL-18’ or ‘interleukin 18’ or ‘IL 18’ or ‘cytotoxic T lymphocyte antigen-4’ or ‘CTLA-4’) and (‘polymorphism’ or ‘variant’ or ‘variation’ or ‘mutation’ or ‘SNP’ or ‘genome-wide association study’ or ‘genetic association study’ or ‘genotype’ or ‘allele’) and (‘viral hepatitis’ or ‘chronic hepatitis’ or ‘acute hepatitis’ or ‘Hepatitis A’ or ‘Hepatitis B’ or ‘Hepatitis C’ or ‘Hepatitis D’ or ‘Hepatitis E’ or ‘HAV’ or ‘HBV’ or ‘HCV’ or ‘HDV’ or ‘HEV’). The references of retrieved articles were also screened by us in case some related articles may be missed by our electronic literature searching.

To be included in this meta-analysis, some criteria must be satisfied: (I) studies about associations between polymorphisms in *CTLA-4*/*IL-18* and viral hepatitis in humans; (II) offer genotypic or allelic distribution of *CTLA-4/IL-18* polymorphisms in patients with viral hepatitis and controls and (III) full manuscript in English or Chinese is retrievable. We only included the most up-to-date study if duplicate reports were found during literature search.

### Data extraction and quality assessment

Two authors extracted following information from eligible articles: (I) name of the first author; (II) published year; (III) country where the study was conducted; (IV) ethnicity of involved participants; (V) number of patients with viral hepatitis and controls in each study and (VI) genotype distributions of polymorphisms in *CTLA-4/IL-18* among patients with viral hepatitis and controls. *P* Values of Hardy–Weinberg equilibrium (HWE) were also calculated.

The authors used Newcastle–Ottawa scale (NOS) to assess the methodology quality of eligible articles [10]. The score range of NOS is between zero and nine, when an article got a score of seven or more, we considered that the methodology of this publication was good.

Two authors extracted data and assessed the quality of eligible articles. The authors wrote to the corresponding authors for additional information if essential information was found to be incomplete.

### Statistical analyses

We used Review Manager to combine the results of individual studies. The *Z* test was employed to assess associations between polymorphisms in *CTLA-4/IL-18* and susceptibility to viral hepatitis in dominant, recessive, over-dominant and allele comparisons. All *CTLA-4/IL-18* polymorphisms contain a major allele (M) and a minor allele (m), the dominant comparison is defined as MM *vs.* Mm + mm, recessive comparison is defined as mm *vs.* MM + Mm, over-dominant comparison is defined as Mm *vs.* MM + mm and the allele comparison is defined as M *vs.* m. The statistical significant threshold of the *P* value was set at 0.05. *I*^2^ statistics were used to assess between-study heterogeneities. Random-effect models (DerSimonian–Laird method) were used to combine the results if *I*^2^ is larger than 50%. Otherwise, fixed-effect models (Mantel–Haenszel method) were used to combine the results. We also carried out subgroup analyses first by type of disease and then by ethnicity of participants. We examined the stability of combined results by deleting one study each time and combining the results of the remaining studies. Funnel plots were used to estimate whether our combined results may be influenced by overt publication biases.

## Results

### Characteristics of included studies

**Fig. 1.**
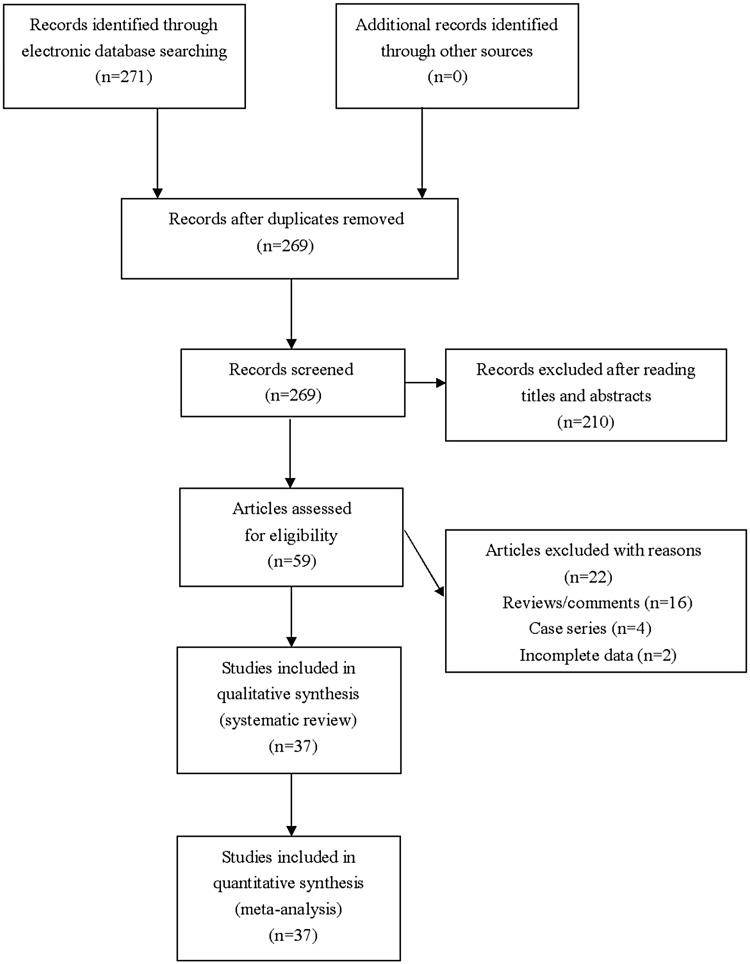
Flowchart of study selection for the present study.

We identified 271 articles during literature searching. Fifty-nine articles were assessed for eligibility after excluding unrelated or duplicate publications. We further excluded 16 reviews and four case controls, and another two articles were excluded because of missing crucial data. Totally 37 articles were ultimately included in this meta-analysis (Fig. 1). Extracted data of eligible articles are shown in Table 1.

**Table 1.** The characteristics of included studies for this meta-analysis

First author, year	Country	Ethnicity	Type of disease	Sample size	Genotype distribution		*P*-Value for HWE	NOS score
					Cases	Controls		
*CTLA-4* rs231775								
Chen 2014	China	East Asian	HBV	465/204	198/214/53	82/102/20	0.146	8
Danilovic 2012	Brazil	Mixed	HCV	112/183	59/34/19	81/67/35	0.003	7
Duan 2011	China	East Asian	HBV	172/145	50/89/33	61/68/16	0.648	7
Enciso-Vargas 2018	México	Mixed	HCV	205/215	67/104/34	93/89/33	0.134	7
Gu 2010	China	East Asian	HBV	570/407	244/251/75	183/179/45	0.902	8
Jiang 2007	China	East Asian	HBV	24/143	5/15/4	70/61/12	0.800	7
Khorshied 2014	Egypt	South Asian	HCV	52/460	28/40/5	203/204/53	0.872	7
Ksiaa 2015	Tunisia	South Asian	HCV	500/358	225/198/77	176/142/40	0.168	7
Mohammad 2006	Iran	South Asian	HBV	51/150	9/16/26	41/52/57	<0.001	8
Sepahi 2017	Iran	South Asian	HCV	65/65	NA	NA	NA	7
Thio 2004	USA	Mixed	HBV	378/676	NA	NA	NA	7
Xiao 2015	China	East Asian	HCV	816/375	358/375/83	176/168/31	0.300	8
Zhang 2012	China	East Asian	HBV	172/145	50/89/33	61/68/16	0.648	8
*CTLA-4* rs5742909								
Chen 2014	China	East Asian	HBV	464/200	342/112/10	160/38/2	0.877	8
Danilovic 2012	Brazil	Mixed	HCV	112/183	92/19/1	152/31/0	0.211	7
Duan 2011	China	East Asian	HBV	172/145	141/28/3	105/39/1	0.194	7
Enciso-Vargas 2018	México	Mixed	HCV	205/215	183/22/0	193/21/1	0.604	7
Khorshied 2014	Egypt	South Asian	HCV	54/503	33/13/8	403/67/29	<0.001	7
Mohammad 2006	Iran	South Asian	HBV	51/150	41/10/0	134/16/0	0.492	8
Schott 2007	Germany	Caucasian	HBV	323/202	276/42/5	150/47/5	0.570	7
Sepahi 2017	Iran	South Asian	HCV	65/65	55/10/0	55/9/1	0.392	7
Thio 2004	USA	Mixed	HBV	378/676	NA	NA	NA	7
Xiao 2015	China	East Asian	HCV	816/375	523/269/24	266/99/10	0.829	8
Zhang 2012	China	East Asian	HBV	172/145	141/28/3	105/39/1	0.194	8
*CTLA-4* rs3087243								
Chen 2014	China	East Asian	HBV	467/203	301/148/18	116/79/8	0.223	8
Danilovic 2012	Brazil	Mixed	HCV	112/183	38/53/21	62/95/26	0.279	7
Ksiaa 2015	Tunisia	South Asian	HCV	500/358	194/217/89	124/158/76	0.056	7
Thio 2004	USA	Mixed	HBV	378/676	NA	NA	NA	7
Xiao 2015	China	East Asian	HCV	816/375	555/231/30	266/99/10	0.829	8
*IL-18* rs1946518								
Abdelraheem 2016	Egypt	South Asian	HCV	100/100	21/47/32	42/51/7	0.104	7
An 2008	USA	Caucasian	HCV	384/212	NA	NA	NA	7
An 2008	USA	African	HCV	364/182	NA	NA	NA	7
Bakr 2018	Egypt	South Asian	HCV	189/90	30/79/80	24/48/18	0.498	8
Bao 2015	China	East Asian	HBV	153/165	37/73/43	41/76/48	0.322	8
Bouzgarrou 2008	Tunisia	South Asian	HCV	81/82	24/38/19	21/44/17	0.493	8
Cheong 2010	South Korea	East Asian	HBV	696/313	183/321/192	87/148/78	0.344	7
Dai 2017	China	East Asian	HBV	250/250	61/134/55	64/124/62	0.900	8
Estfanous 2019	Egypt	South Asian	HCV	201/95	70/92/39	47/37/11	0.378	8
Falleti 2007	Italy	Caucasian	HCV	46/105	12/22/12	33/42/30	0.041	8
Haas 2009	Germany	Caucasian	HCV	757/791	276/347/134	300/369/122	0.628	8
Hirankarn 2007	Thailand	East Asian	HBV	140/140	33/68/39	39/83/18	0.012	8
Imran 2014	Pakistan	South Asian	HCV	140/120	25/50/55	35/53/32	0.203	7
Karra 2015	India	South Asian	HBV	271/280	70/152/49	102/144/34	0.120	8
Ksiaa 2011	Tunisia	South Asian	HCV	100/100	30/44/26	26/50/24	0.997	8
Li 2012	China	East Asian	HBV	501/301	121/239/141	85/156/60	0.448	7
Lu 2015	China	East Asian	HBV	129/160	32/58/39	40/73/47	0.278	8
Mandour 2014	Egypt	South Asian	HCV	123/123	20/63/40	26/58/39	0.608	8
Migita 2009	Japan	East Asian	HBV	204/63	55/119/30	20/30/13	0.777	8
Santos 2015	Brazil	Mixed	HCV	304/376	36/156/112	68/192/116	0.459	8
Wu 2011	China	East Asian	HBV	12/109	3/8/1	37/46/26	0.124	7
Yue 2013	China	East Asian	HCV	552/784	NA	NA	NA	7
Zhang 2005	China	East Asian	HBV	231/300	53/116/62	74/160/66	0.243	8
*IL-18* rs187238								
An 2008	USA	Caucasian	HCV	384/212	NA	NA	NA	7
An 2008	USA	African	HCV	364/182	NA	NA	NA	7
Bakr 2018	Egypt	South Asian	HCV	189/90	99/87/3	30/58/2	<0.001	8
Bao 2015	China	East Asian	HBV	153/165	122/28/3	106/54/5	0.548	8
Bouzgarrou 2008	Tunisia	South Asian	HCV	81/82	38/31/12	35/35/12	0.506	8
Cheong 2010	South Korea	East Asian	HBV	707/316	546/155/6	237/67/12	0.013	7
Dai 2017	China	East Asian	HBV	250/250	200/48/2	183/65/2	0.142	8
Estfanous 2019	Egypt	South Asian	HCV	201/95	102/94/5	52/36/7	0.824	8
Falleti 2007	Italy	Caucasian	HCV	50/96	23/23/4	49/38/9	0.681	8
Haas 2009	Germany	Caucasian	HCV	757/791	386/315/56	439/299/53	0.829	8
Hirankarn 2007	Thailand	East Asian	HBV	140/140	105/29/6	102/35/3	0.999	8
Imran 2014	Pakistan	South Asian	HCV	140/120	57/70/13	43/61/16	0.437	7
Jiang 2014	China	East Asian	HBV	276/254	221/51/4	168/80/6	0.324	7
Karra 2015	India	South Asian	HBV	271/280	123/134/14	159/108/13	0.320	8
Ksiaa 2011	Tunisia	South Asian	HCV	100/100	53/33/14	44/44/12	0.845	8
Lu 2015	China	East Asian	HBV	129/160	100/27/2	103/52/5	0.610	8
Migita 2009	Japan	East Asian	HBV	204/63	167/32/5	52/10/1	0.531	8
Santos 2015	Brazil	Mixed	HCV	304/376	100/120/84	128/132/116	<0.001	8
Wu 2011	China	East Asian	HBV	12/109	11/1/0	85/22/2	0.682	7
Yue 2013	China	East Asian	HCV	552/784	NA	NA	NA	7
Zhang 2005	China	East Asian	HBV	231/300	182/45/4	202/90/8	0.588	8

CTLA-4, cytotoxic T-lymphocyte-associated antigen 4; IL-6, interleukin-6; HBV, hepatitis B virus infection; HCV, hepatitis C virus infection; HWE, Hardy–Weinberg equilibrium; NOS, Newcastle–Ottawa scale; NA, not available.

### Meta-analysis results for polymorphisms in CTLA-4 and viral hepatitis

Fourteen eligible articles were about *CTLA-4* polymorphisms and viral hepatitis. *CTLA-4* rs231775 (recessive comparison: OR 1.33, 95% CI 1.23–1.43) polymorphism was found to be significantly associated with viral hepatitis in overall combined analyses. Further subgroup analyses revealed similar positive findings for *CTLA-4* rs231775 polymorphism in HBV, especially among East Asians. A significant association with HCV was also detected for *CTLA-4* rs5742909 polymorphism (see Table 2).

**Table 2.** Meta-analysis results of this study

Variables	Sample size	Dominant comparison		Recessive comparison		Over-dominant comparison		Allele comparison	
		*P* value	OR (95% CI)	*P* value	OR (95% CI)	*P* value	OR (95% CI)	*P* value	OR (95% CI)
*CTLA-4* rs231775									
Overall	3582/3526	0.05	0.83 (0.69–1.00)	**0.001**	**1.31 (1.11–1.55)**	0.16	1.15 (0.95–1.40)	0.14	0.88 (0.74–1.04)
HBV	1832/1870	**0.03**	**0.70 (0.51–0.96)**	**0.002**	**1.46 (1.15–1.86)**	0.68	1.03 (0.88–1.22)	**0.0007**	**0.75 (0.63–0.88)**
East Asian	1403/1044	0.06	0.71 (0.51–1.12)	**0.006**	**1.43 (1.11–1.85)**	0.60	1.05 (0.89–1.24)	**0.02**	**0.76 (0.60–0.96)**
HCV	1750/1656	0.64	0.94 (0.73–1.21)	0.14	1.19 (0.94–1.49)	0.51	1.05 (0.90–1.23)	0.07	0.90 (0.80–1.01)
South Asian	617/883	0.85	0.95 (0.62–1.79)	0.14	1.32 (0.91–1.91)	0.34	1.97 (0.48–3.07)	0.28	0.55 (0.19–1.61)
*CTLA-4* rs5742909									
Overall	2812/2859	0.80	0.96 (0.68–1.34)	0.12	1.43 (0.91–2.25)	1.00	1.00 (0.72–1.39)	0.93	0.99 (0.75–1.30)
HBV	1560/1518	0.47	1.21 (0.72–2.04)	0.42	1.39 (0.63–3.07)	0.38	0.79 (0.47–1.34)	0.25	1.21 (0.87–1.67)
East Asian	808/490	0.49	1.26 (0.66–2.38)	0.13	2.35 (0.77–7.12)	0.37	0.74 (0.38–1.44)	0.62	1.14 (0.67–1.95)
HCV	1252/1341	**0.003**	**0.73 (0.60–0.90)**	0.18	1.46 (0.84–2.52)	**0.01**	**1.32 (1.07–1.64)**	0.08	0.75 (0.54–1.03)
South Asian	119/568	0.24	0.58 (0.23–1.44)	0.05	2.25 (0.99–5.09)	0.08	1.66 (0.94–2.91)	0.34	0.63 (0.24–1.64)
*CTLA-4* rs3087243									
Overall	2273/1795	0.35	1.08 (0.92–1.26)	0.84	0.97 (0.75–1.27)	0.42	0.94 (0.80–1.10)	0.74	0.97 (0.78–1.19)
HBV	845/879	0.07	1.36 (0.97–1.90)	0.96	0.98 (0.42–2.29)	0.07	0.73 (0.52–1.03)	0.85	0.95 (0.58–1.57)
HCV	1428/916	0.91	1.01 (0.84–1.21)	0.84	0.97 (0.74–1.28)	0.99	1.00 (0.84–1.20)	0.94	0.99 (0.81–1.22)
*IL-18* rs1946518									
Overall	6270/5800	**<0.0001**	**0.82 (0.75–0.90)**	**0.0008**	**1.29 (1.11–1.50)**	0.49	0.97 (0.89–1.05)	**<0.0001**	**0.76 (0.68–0.86)**
HBV	2587/2081	**0.01**	**0.84 (0.73–0.96)**	0.10	1.20 (0.97–1.49)	0.91	0.99 (0.88–1.12)	**0.009**	**0.82 (0.70–0.95)**
East Asian	2316/1801	0.11	0.89 (0.77–1.03)	0.21	1.16 (0.92–1.47)	0.59	0.97 (0.85–1.10)	**0.03**	**0.83 (0.71–0.98)**
HCV	3683/3719	**0.004**	**0.73 (0.59–0.91)**	**0.003**	**1.38 (1.11–1.71)**	0.39	0.95 (0.85–1.07)	**0.0008**	**0.73 (0.60–0.88)**
South Asian	1486/1494	**0.001**	**0.65 (0.52–0.81)**	**0.008**	**1.65 (1.14–2.38)**	0.18	0.87 (0.71–1.06)	**0.004**	**0.67 (0.51–0.88)**
*IL-18* rs187238									
Overall	5495/4965	**0.03**	**1.25 (1.03–1.52)**	0.12	0.86 (0.71–1.04)	0.08	0.85 (0.71–1.02)	**0.007**	**1.20 (1.05–1.37)**
HBV	2373/2037	**0.03**	**1.39 (1.04–1.86)**	0.11	0.72 (0.48–1.07)	**0.04**	**0.73 (0.54–0.98)**	**0.02**	**1.33 (1.05–1.70)**
East Asian	2102/1757	**<0.0001**	**1.53 (1.32–1.79)**	**0.04**	**0.61 (0.39–0.98)**	**<0.0001**	**0.67 (0.58–0.79)**	**<0.0001**	**1.47 (1.28–1.68)**
HCV	3122/2928	0.49	1.08 (0.86–1.36)	0.37	0.91 (0.73–1.12)	0.97	1.00 (0.83–1.22)	0.36	1.04 (0.95–1.14)
South Asian	510/392	**0.004**	**1.49 (1.13–1.95)**	0.65	0.90 (0.57–1.42)	**0.009**	**0.70 (0.53–0.91)**	**0.02**	**1.27 (1.04–1.55)**

OR, odds ratio; CI, confidence interval; NA, not available; HBV, hepatitis B virus infection; HCV, hepatitis C virus infection.

The values in bold represent there is statistically significant differences between cases and controls.

### Meta-analysis results for polymorphisms in IL-18 and viral hepatitis

Twenty-three articles were about *IL-18* polymorphisms and viral hepatitis. *IL-18* rs1946518 (dominant comparison: OR 0.82, 95% CI 0.75–0.90; recessive comparison: OR 1.29, 95% CI 1.11–1.50; allele comparison: OR 0.76, 95% CI 0.68–0.86) and *IL-18* rs187238 (dominant comparison: OR 1.25, 95% CI 1.03–1.52; allele comparison: OR 1.20, 95% CI 1.05–1.37) polymorphisms were both found to be significantly associated with viral hepatitis in overall combined analyses. Further subgroup analyses revealed similar positive findings for *IL-18* rs1946518 and rs187238 polymorphisms in HBV, especially among East Asians. Moreover, we also found that *IL-18* rs1946518 and rs187238 polymorphisms were significantly associated with susceptibility to HCV, especially among South Asians (see Table 2).

### Sensitivity analyses

Stabilities of combined results were examined by deleting one study each time and combining the results of the remaining studies. The trends of associations remained consistent in sensitivity analyses, indicating that the combined results were statistically stable.

### Publication biases

Funnels plots were employed to estimate whether our combined results may be influenced by overt publication biases. Funnel plots were overall symmetrical, indicating that the combined results were unlikely to be seriously impacted by overt publication biases (see Supplementary Fig. S1).

## Discussion

The combined results of this meta-analysis revealed that *CTLA-4* rs231775, *IL-18* rs1946518 and *IL-18* rs187238 polymorphisms were significantly associated with susceptibility to HBV, especially among East Asians. Moreover, *CTLA-4* rs5742909, *IL-18* rs1946518 and *IL-18* rs187238 polymorphisms were also found to be significantly associated with susceptibility to HCV, especially among South Asians. The trends of associations remained consistent in sensitivity analyses, indicating that the combined results were stable.

Some points need to be considered when interpreting our findings. First, past pre-clinical studies found that rs231775, rs5742909 and rs3087243 polymorphisms in *CTLA-4* as well as rs1946518 and rs187238 polymorphisms in *IL-18* could alter transcription activity or protein structure of *CTLA-4/IL-18* [11–14]. So these variations may influence biological function of *CTLA-4/IL-18*, result in immune dysfunction, impact anti-viral immune responses and ultimately confer susceptibility to viral hepatitis. Thus, our meta-analysis may be statistically insufficient to observe the real underlying associations between polymorphisms in *CTLA-4/IL-18* and viral hepatitis in certain groups. Therefore, future studies with larger sample sizes still need to confirm our findings. Second, according to our searching results, studies about HBV were mainly conducted in East Asians, whereas studies in HCV were mainly conducted in South Asians. So we call on scholars to examine associations between polymorphisms in *CTLA-4/IL-18* and viral hepatitis in other populations. Third, the etiologies of viral hepatitis are extremely complex, so we highly recommend further genetic association studies to explore the effects of haplotypes and gene–gene interactions on disease susceptibility [15]. Fourth, we aimed to investigate associations between all polymorphisms in *CTLA-4/IL-18* and viral hepatitis in the very beginning. However, we did not find any study on other *CTLA-4/IL-18* polymorphisms. Nor did we find any studies about HAV, HDV or HEV. So we only focused on associations of five polymorphisms with HBV and HCV in this meta-analysis.

Like all meta-analyses, this study also has some limitations. Firstly, the results regarding associations between polymorphisms in *CTLA-4/IL-18* and viral hepatitis were based on combining unadjusted findings of eligible studies due to the lack of raw data [16]. Secondly, the relationship between polymorphisms in *CTLA-4/IL-18* and viral hepatitis may also be affected by environmental factors. Nevertheless, the majority of eligible studies only focused on associations between polymorphisms in *CTLA-4/IL-18* and viral hepatitis, so we could not explore genetic-environmental interactions in this meta-analysis [17]. Thirdly, grey literatures were not searched. Thus, despite that funnel plots were overall symmetrical, we still could not rule out the possibility that our combined results may be affected by potential publication biases [18].

## Conclusions

In summary, this meta-analysis demonstrated that *CTLA-4* rs231775, *IL-18* rs1946518 and *IL-18* rs187238 polymorphisms may confer susceptibility to HBV in East Asians, while *CTLA-4* rs5742909, *IL-18* rs1946518 and *IL-18* rs187238 polymorphisms may confer susceptibility to HCV in South Asians. However, it should be noted that the combined results of this meta-analysis should still be confirmed by future studies with larger sample sizes.

## Supplementary material

For supplementary material accompanying this paper visit https://doi.org/10.1017/S0950268819001997. click here to view supplementary material

## Data

Data sharing is not applicable to this article as no new data were created or analysed in this study.
