# Supplementary material for: Relationship of genetic polymorphisms in CTLA-4 and IL-18 with viral hepatitis: evidence from a meta-analysis
Source: Epidemiol Infect. 2019 Dec 5;147:e313. doi: 10.1017/S0950268819001997 (PMC7003626; doi:10.1017/S0950268819001997)
Supplement: Supplementary file 1 [file S0950268819001997sup001.docx]

**Supplementary Figure 1. Funnel plots of investigated polymorphisms**


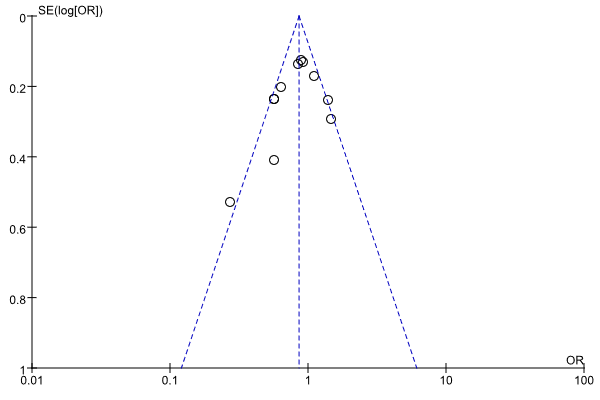


Funnel plot of CTLA-4 rs231775 polymorphism and viral hepatitis under dominant comparison


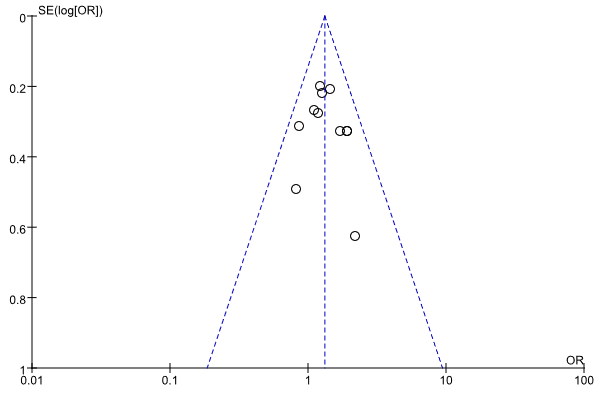


Funnel plot of CTLA-4 rs231775 polymorphism and viral hepatitis under recessive comparison


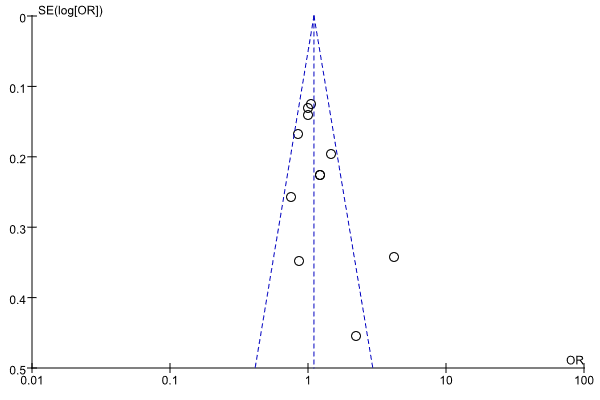


Funnel plot of CTLA-4 rs231775 polymorphism and viral hepatitis under over-dominant comparison


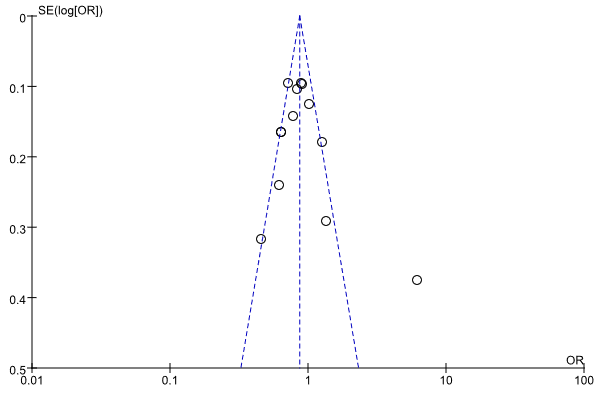


Funnel plot of CTLA-4 rs231775 polymorphism and viral hepatitis under allele comparison


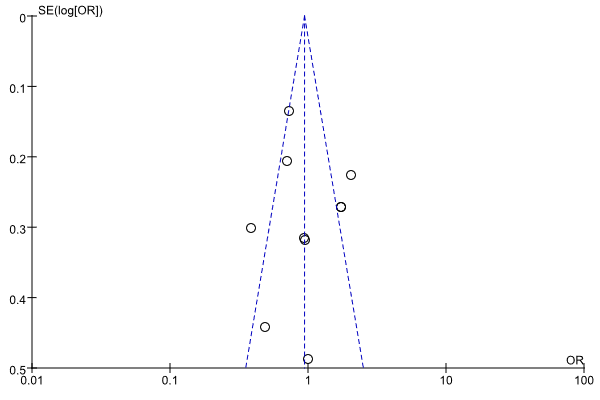


Funnel plot of CTLA-4 rs5742909 polymorphism and viral hepatitis under dominant comparison


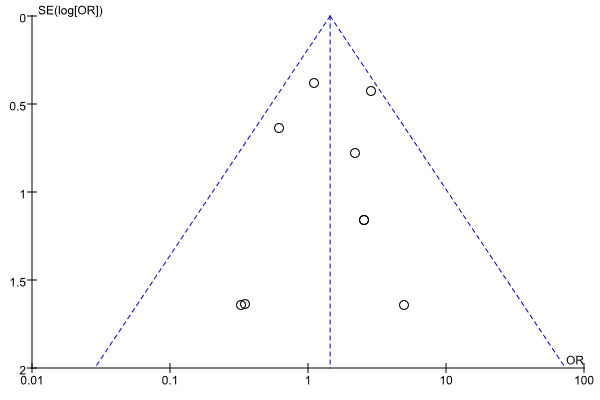


Funnel plot of CTLA-4 rs5742909 polymorphism and viral hepatitis under recessive comparison


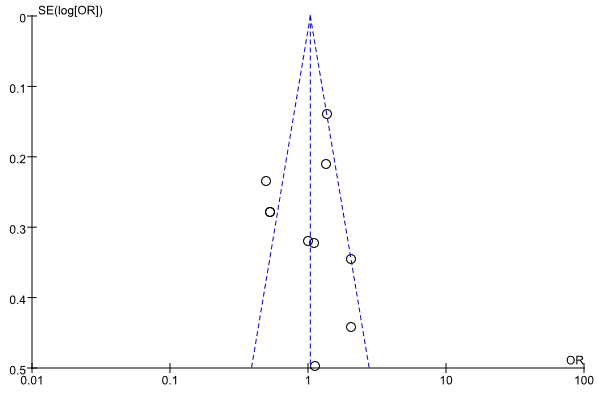


Funnel plot of CTLA-4 rs5742909 polymorphism and viral hepatitis under over-dominant comparison


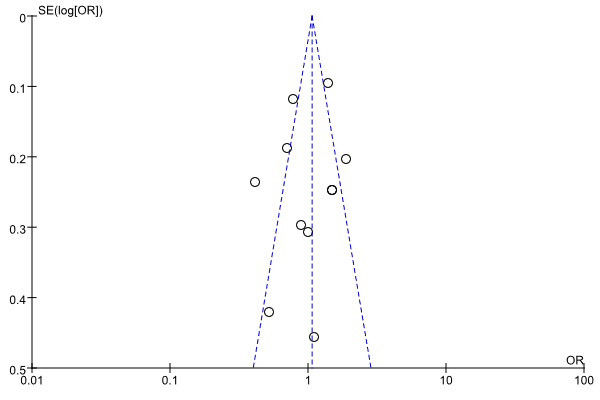


Funnel plot of CTLA-4 rs5742909 polymorphism and viral hepatitis under allele comparison


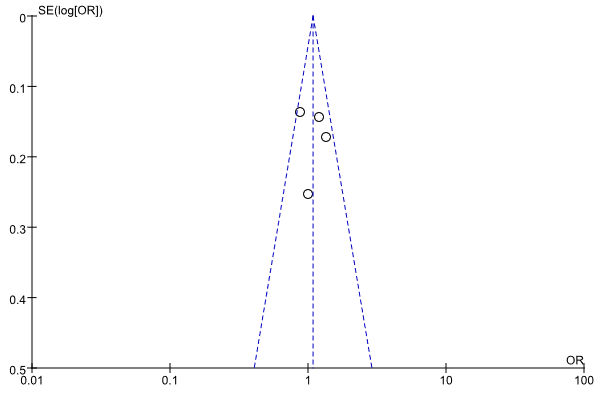


Funnel plot of CTLA-4 rs3087243 polymorphism and viral hepatitis under dominant comparison


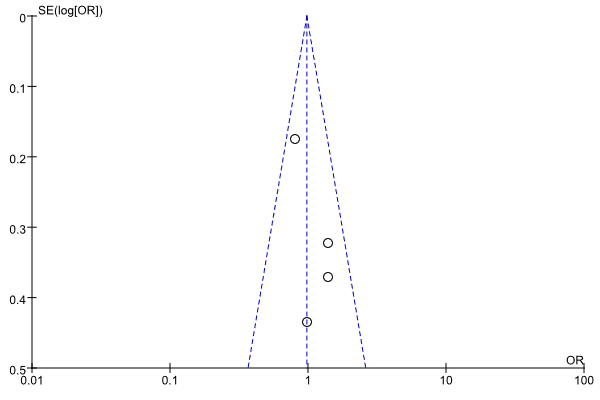


Funnel plot of CTLA-4 rs3087243 polymorphism and viral hepatitis under recessive comparison


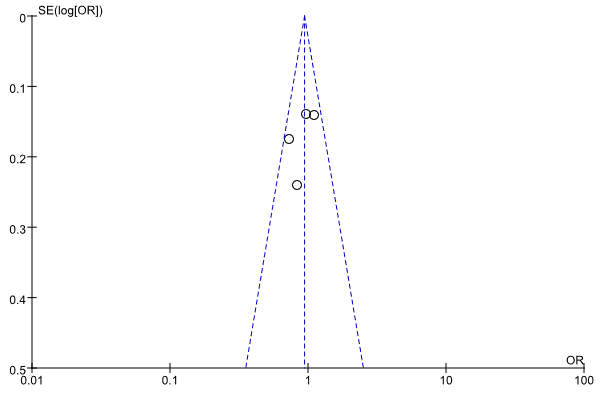


Funnel plot of CTLA-4 rs3087243 polymorphism and viral hepatitis under over-dominant comparison


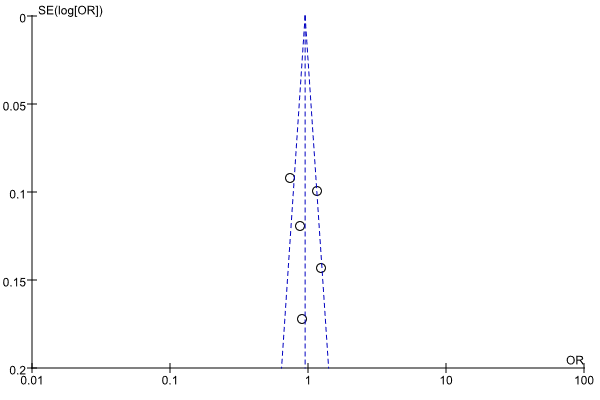


Funnel plot of CTLA-4 rs3087243 polymorphism and viral hepatitis under allele comparison


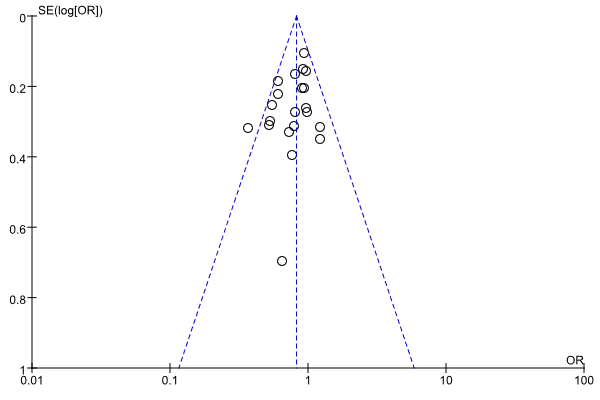


Funnel plot of IL-18 rs1946518 polymorphism and viral hepatitis under dominant comparison


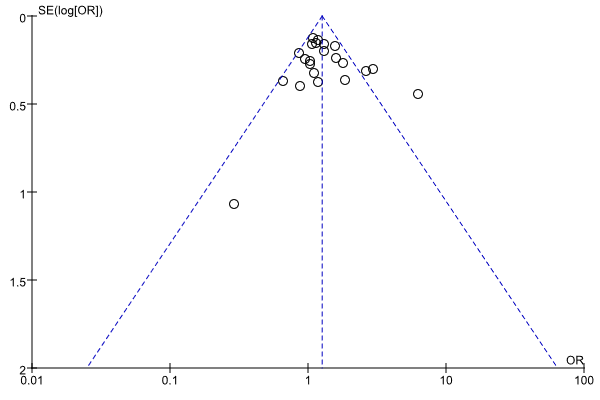


Funnel plot of IL-18 rs1946518 polymorphism and viral hepatitis under recessive comparison


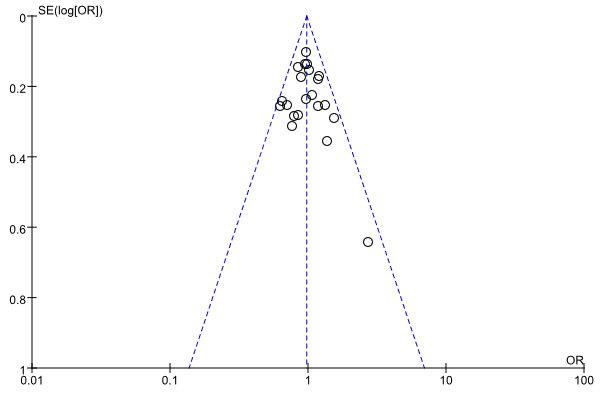


Funnel plot of IL-18 rs1946518 polymorphism and viral hepatitis under over-dominant comparison


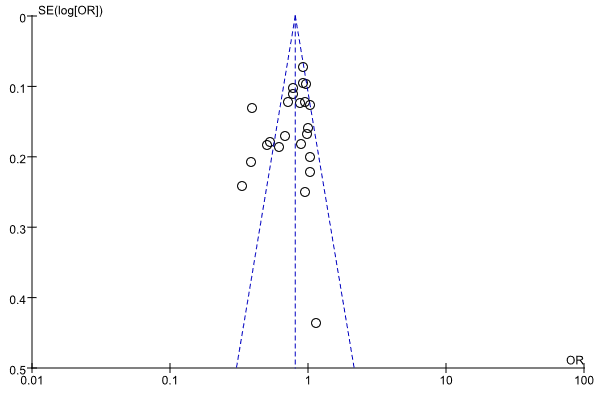


Funnel plot of IL-18 rs1946518 polymorphism and viral hepatitis under allele comparison


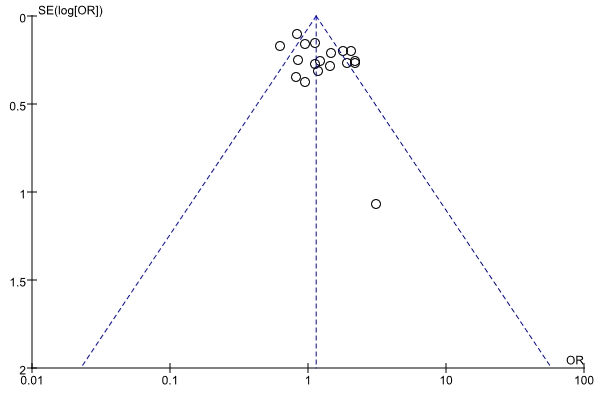


Funnel plot of IL-18 rs187238 polymorphism and viral hepatitis under dominant comparison


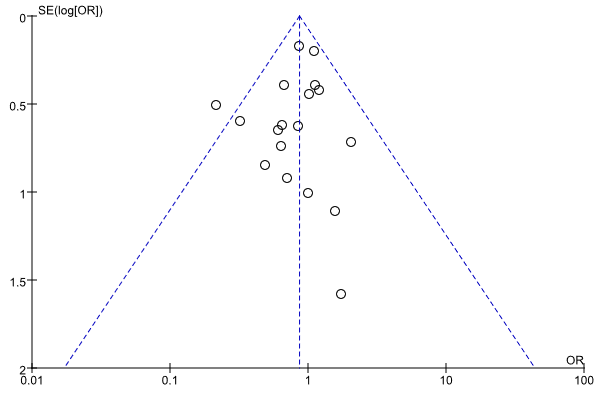


Funnel plot of IL-18 rs187238 polymorphism and viral hepatitis under recessive comparison


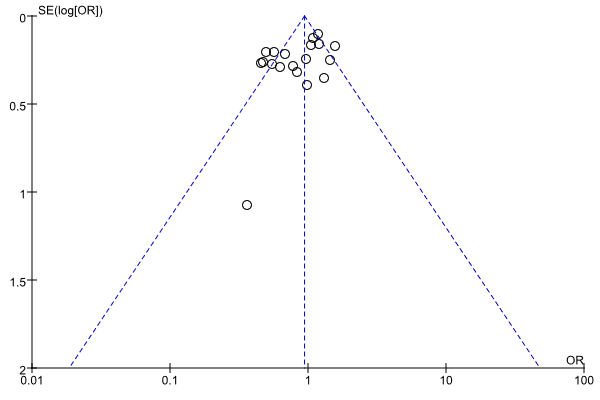


Funnel plot of IL-18 rs187238 polymorphism and viral hepatitis under over-dominant comparison


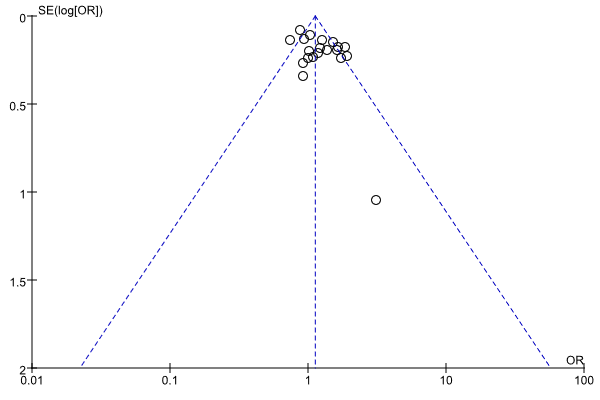


Funnel plot of IL-18 rs187238 polymorphism and viral hepatitis under allele comparison
